# Notes and News

**Published:** 1904-05-14

**Authors:** 


### Notes and News.

The Prince and Princess of Wales, Grand President and Lady Grand President of the League of Mercy, have graciously consented to receive the Presidents, Lady President, and certain others connected with the League at Marlborough House on the afternoon of Wednesday, June 8th.

The annual meeting of the Hospital for Sick Children, Great Ormond Street, will be held on May 26th, at 5 p.m., the Duke of Fife presiding.

A new nurses' home has been opened at Chatham in connection with the Chatham Nursing Association, affiliated to Queen Victoria's Jubilee Institute. The site was given by Mrs. Mary Winch, and the building, costing £1,000, constitutes Chatham's memorial to Queen Victoria.

Lecturing before the Ambidextral Culture Society recently, the Rev. H. Dukinfield Astley remarked that there was evidence that neolithic man used his left hand very largely, and early Greek and Roman inscriptions written from left to right show that with them ambidexterity was still practised in writing.

A special general meeting of the Governors and subscribers of St. John's Hospital for Diseases of the Skin, under the presidency of the Earl of Chesterfield, to confirm the resolutions to incorporate the hospital passed at a previous meeting, will be held on Tuesday, May 31st, at 4.30, at the Whitehall Rooms, Hôtel Métropole.

A deputation representing the new Street Ambulance Association waited on the General Purposes Committee of the London County Council last week with a view of inducing the Council to take the matter up. Amongst the deputation were representatives of St. John's Ambulance Association, which is favourable to the movement. It was also stated that Mr. Bischoffsheim had written in sympathy with the aims of the new association, and expressing his regret that he was unable to join the deputation. The deputation of the Metropolitan Street Ambulance Association, explained the aims of the association, which are to generally improve the existing ambulance arrangements, and as a result of their efforts anticipate that the County Council will take the matter up.

The new Hawick Joint Fever Hospital is now ready for occupation. It contains 24 beds, and was established for an expenditure of £5,000.

Lord Wolverton has promised to preside at the Festival Dinner at the Whitehall Rooms, on June 23rd next, in aid of the funds of the Metropolitan Hospital.

An infectious diseases hospital has been opened for the Rural and Urban District of South Rotherham, Handsworth, and Kiveton Park, near Sheffield. The accommodation is for 32 patients.

Mr. T. C. Langdon, who during 40 years has acted as surgeon of the Royal Hants County Hospital, has been presented with a service of plate, in recognition of his labours on behalf of the institution.

The Royal Southern Hospital, Liverpool, is now in possession of an up-to-date $x$-ray room, which has been enlarged through the generosity of Mr. John W. Hughes. Messrs. Hewitt, of Dedham, have presented a Finsen Light installation to the Colchester Hospital.

A fête and fancy fair in aid of the League of Mercy will be held at Buckhurst, Wokingham, the residence of Mrs. Murdoch, towards the end of July, under the presidency of her Highness Princess Victoria of Schleswig-Holstein, Lady President of the League of Mercy for Berkshire. Their Royal Highnesses Prince and Princess Christian have also notified their intention of being present.

Mr. Arthur Chamberlain last week laid the foundation-stone of a new women's hospital to take the place of the old Birmingham and Midland Hospital for Women. The new building, which is to be of two storeys, is intended for 48 patients. A narrow balcony will run along the sides of the wards on the first floor. The nurses' home is detached, as is also the laundry and engineering department and mortuary, but there is access through a subway. A convalescent home is part of the scheme. The estimate is for £40,000, towards which £32,000 has already been raised.

The consideration of the Bill authorising the removal of King's College Hospital from Lincoln's Inn Fields to Camberwell has been concluded and the Bill was ordered to be reported to the House of Lords for the third reading. The following clause, relating to the Pest House Charity, by which the hospital has benefited for many years was inserted:—"At any time after the removal of the hospital from the present site the trustees of the charity known as the Pest House Charity may, if they think fit, give notice thereof to the Charity Commissioners, who may, if they think fit, settle a new scheme for the administration of the said charity, but nothing in this section contained shall in any way interfere with the discretion of the Charity Commissioners as to the continuance of the present scheme or the settlement of a new scheme." The Pest House Charity provides certain benefits for inhabitants of Westminster, and therefore it is argued that King's College cannot appropriate the charity as a matter of course after removal.

